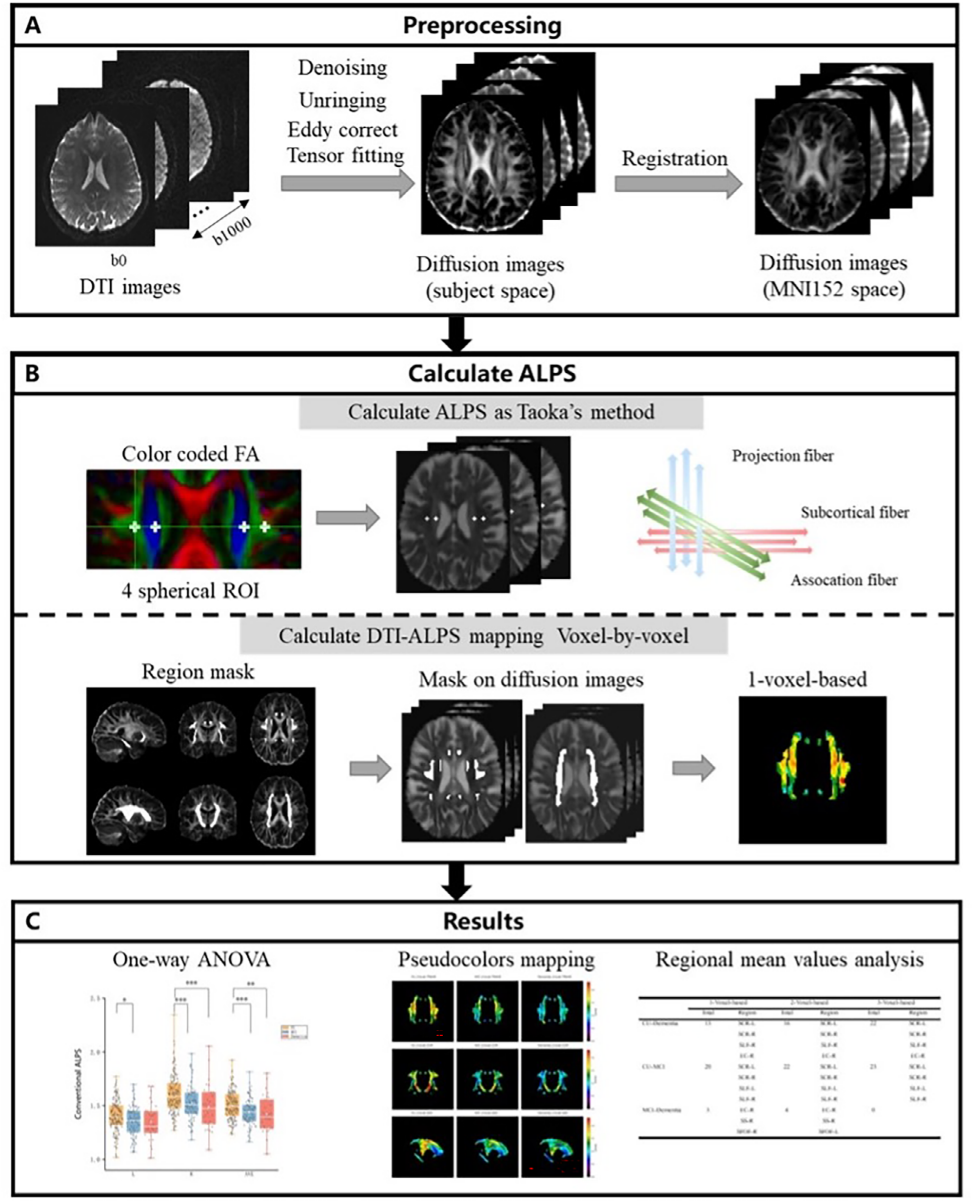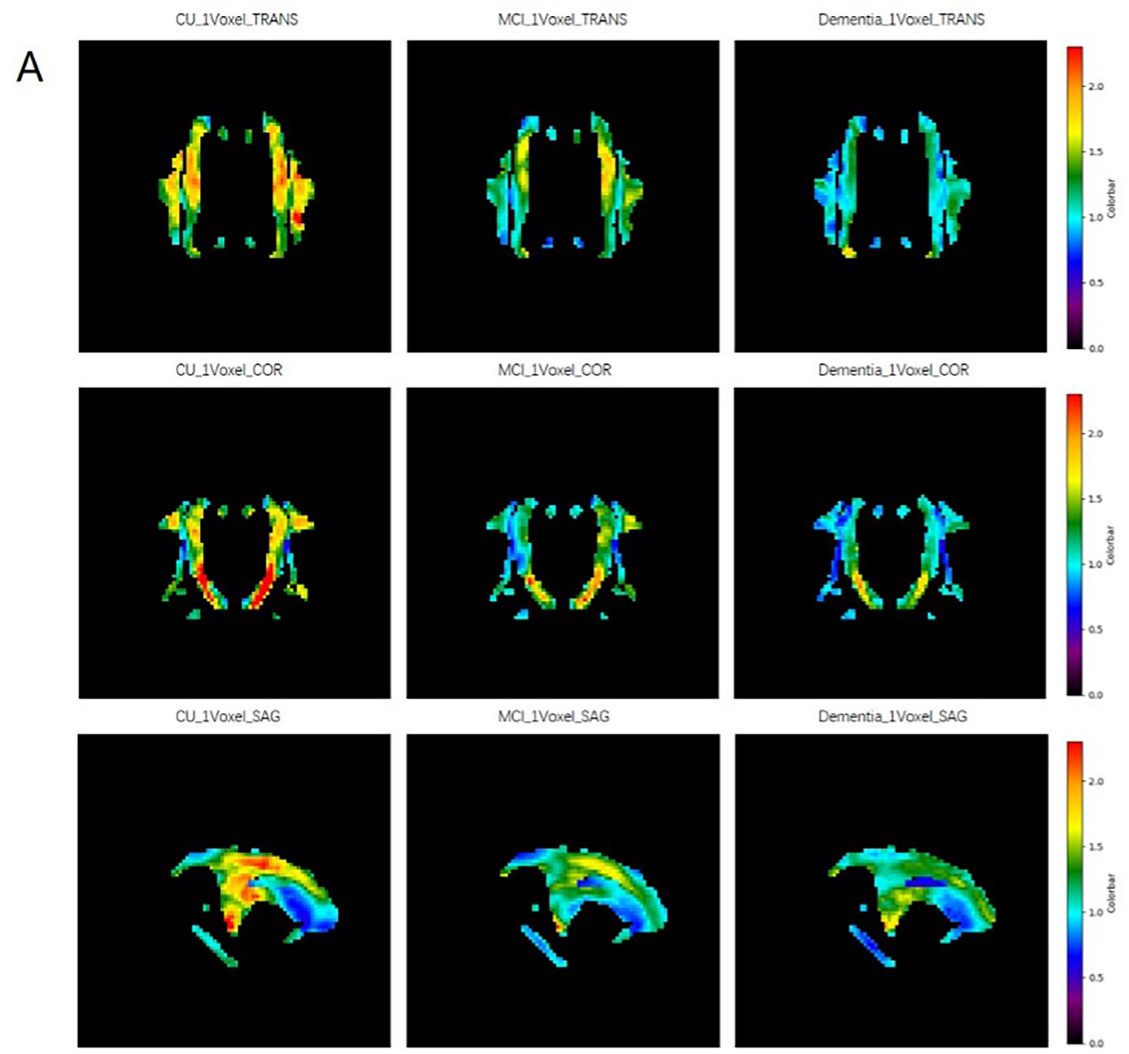# DTI‐ALPS Mapping: A Novel Voxel‐based Method for Analyzing the Glymphatic System in the Brain

**DOI:** 10.1002/alz70862_109947

**Published:** 2025-12-23

**Authors:** Xiang Fan, Xiqian Zhang, Keyan Yu, Lele Chen, Zhuonan Wei, Gaigai Lu, Hui Chen, Lin Hu, Guanxun Cheng, Na Zhang

**Affiliations:** ^1^ Peking University Shenzhen Hospital, Shenzhen, Guangdong China; ^2^ Shenzhen Institute of Advanced Technology, Chinese Academy of Sciences, Shenzhen, Guangdong China

## Abstract

**Background:**

The glymphatic system is increasingly recognized as a critical factor in the pathogenesis of dementia, which can be assessed using the conventional diffusion tensor image analysis along the perivascular space (DTI‐ALPS) based on regions of interest (ROIs). We proposed a novel voxel‐based method to analyze the whole cerebral white matter DTI‐ALPS and named it DTI‐ALPS mapping. The goal of DTI‐ALPS mapping is to characterize changes in whole cerebral white matter DTI‐ALPS patterns in cognitive impairment and to provide a full‐scale, voxel‐basedapproach for assessing glymphatic system activity.

**Method:**

We included 304 participants from the Shenzhen Multimodal Aging Research (STAR) Cohort recruited at Peking University Shenzhen Hospital, including 182 cognitively unimpaired (CU) participants, 93 participants with mild cognitive impairment, and 29 patients with dementia. All participants underwent 3.0T MRI scans with 3D T1‐weighted imaging and DTI sequences. We used DTI‐ALPS mapping to visualize the entire cerebral white matter fibers of 30 regions. We calculated the DTI‐ALPS values using both the conventional ROI‐based method and the voxel‐based DTI‐ALPS mapping we developed. To compare ALPS values among the three groups, we conducted an analysis of variance (ANOVA), followed by post‐hoc tests to identify intergroup differences in both the conventional method and DTI‐ALPS mapping, respectively. We analyzed the correlations between DTI‐ALPS mapping and Mini‐Mental State Examination (MMSE), the Montreal Cognitive Assessment (MoCA), and White Matter Hypointensities (WMH) derived from 3D T1‐weighted imaging.

**Result:**

Using the DTI‐ALPS mapping, we revealed whole cerebral white matter ALPS pattern. Over 13 out of 30 regions exhibited significant differences (*p* < 0.05) in the intergroup analyses, providing additional insights beyond those identified with conventional ROI‐based ALPS. Twenty‐six of 30 regions in the DTI‐ALPS mapping showed a positive correlation with MoCA, and 11 of these regions were also positively correlated with MMSE (Spearman, *p* < 0.05) Moreover, 26 of 30 regions showed a negative correlation with WMH (Spearman, *p* < 0.05).

**Conclusion:**

The DTI‐ALPS mapping provides a robust, intuitive, and comprehensive approach to evaluating the changing pattern of the glymphatic system, overcoming the limitations of conventional ROI‐based ALPS methods and revealing the changing pattern associated with cognition and WMH.